# Outcomes of Predialysis Nephrology Care in Elderly Patients Beginning to Undergo Dialysis

**DOI:** 10.1371/journal.pone.0128715

**Published:** 2015-06-01

**Authors:** Seon Ha Baek, Shin young Ahn, Sung Woo Lee, Youn Su Park, Sejoong Kim, Ki Young Na, Dong-Wan Chae, Suhnggwon Kim, Ho Jun Chin

**Affiliations:** 1 Department of Internal Medicine, Seoul National University Bundang Hospital, Seongnam, Gyeonggi-do, Korea; 2 Department of Internal Medicine, Seoul National University College of Medicine, Seoul, Korea; UNIFESP Federal University of São Paulo, BRAZIL

## Abstract

**Background:**

The proportion of elderly patients beginning to undergo dialysis is increasing globally. Whether early referral (ER) of elderly patients is associated with favorable outcomes remains under debate. We investigated the influence of referral timing on the mortality of elderly patients.

**Methods:**

We retrospectively assessed mortality in 820 patients aged ≥70 years with end-stage renal disease (ESRD) who initiated hemodialysis at a tertiary university hospital between 2000 and 2010. Mortality data was obtained from the time of dialysis initiation until December 2010. We assigned patients to one of two groups according to the time of their first encounters with nephrologists: ER (≥ 3 months) and late referral (LR; < 3 months).

**Results:**

During a mean follow-up period of 25.1 months, the ER group showed a 24% reduced risk of long-term mortality relative to the LR group (HR = 0.760, P = 0.009). Rate of reduction in 90-day mortality for ER patients was 58% (HR = 0.422, P=0.012). However, the statistical significance of the difference in mortality rates between ER and LR group was not observed across age groups after 90 days. Old age, LR, central venous catheter, high white blood cell count and corrected Ca level, and lower levels of albumin, creatinine, hemoglobin, and sodium were significantly associated with increased risk of mortality.

**Conclusions:**

Timely referral was also associated with reduced mortality in elderly ESRD patients who initiated hemodialysis. In particular, the initial 90-day mortality reduction in ER patients contributed to mortality differences during the follow-up period.

## Introduction

The incidence and prevalence of end-stage renal disease (ESRD) have increased worldwide [1,2]. The proportion of elderly patients commencing dialysis is increasing globally [3,4]; however, sizeable variations exist and range from 16% to 40% geographically [5].

Timely referral to a nephrologist has long been recommended in patients with chronic kidney disease (CKD), because late referral (LR) is associated with increased mortality and morbidity and greater healthcare burden [6–8]. Elderly patients with ongoing debate about initiation of dialysis are at risk for experiencing delayed nephrology care [9,10]. While a few studies have reported that LR in the elderly was associated with high mortality rates [11,12], a recent study reported no material improvement in survival rates despite significant trends toward early referral (ER) [13]. Whether ER in the elderly is associated with favorable outcomes remains under debate. Therefore, we investigated predictors of referral timing and the influence of referral timing on the mortality of elderly patients undergoing incident hemodialysis.

## Materials and Methods

### Study population

We included 820 patients aged ≥70 years with ESRD who were receiving incident hemodialysis and had a baseline value of creatinine (Cr) at Seoul National University Hospital in Korea between 2000 and 2010. We excluded patients who were transferred from other chronic dialysis programs, those who experienced acute kidney injury, those for whom the timing of referral was unavailable, or those who underwent peritoneal dialysis as the treatment modality at any given time. This study was approved by the institutional review board of the Seoul National University Hospital (IRB number: H1107-092-370) with no written consent because patients records/information was anonymized and de-identified prior to analysis. All clinical investigations were conducted according to the 2008 Declaration of Helsinki and good clinical practice guidelines.

### Measurements and definitions

Patients’ data were collected retrospectively via review of their electronic medical records. Dates of dialysis initiation were determined using electronic resources at Seoul National University Hospital and the ESRD registry committee of the Korean Society of Nephrology. We adopted a date as that of dialysis initiation when it was registered on two systems, and we thoroughly reviewed medical records when dates differed between the systems. ER and LR were defined according whether the patient’s first encounter with a nephrologist was more than or less than 3 months prior to the first time they were diagnosed with ESRD. Serum Cr values were measured using the alkaline picrate Jaffe kinetic method with an automatic analyzer (Toshiba-200FR, Tokyo, Japan). The estimated glomerular filtration rate (eGFR) was calculated using the Modification of Diet in Renal Disease study equation [14]. Presence of hypertension (HTN) at baseline was confirmed with systolic blood pressure (SBP) of ≥ 140 mmHg or diastolic blood pressure (DBP) of ≥ 90 mmHg during physical examination, a self-reported history of disease, or use of antihypertensive medication. Diabetes mellitus (DM) was confirmed with a glycated hemoglobin level of ≥ 6.5, a self-reported history of disease, or use of antihyperglycemic agents. Cardiovascular disease including angina pectoris (I20), acute myocardial infarction (I21), subsequent myocardial infarction (I22), certain current complications following acute myocardial infarction (I23), other acute ischemic heart disease (I24), chronic ischemic heart disease (I25), heart failure (I50), and cancer (C) at dialysis initiation were specified in the code of the *International Classification of Disease*, *10th Revision* (*ICD-10*).

### Outcome

We combined mortality data after dialysis initiation from Statistics Korea with our dataset, using each individual’s unique identifier. Post ESRD mortality data were obtained until December 2010 [15].

### Statistical analysis

All analyses were conducted using SPSS Statistics V21.0 (IBM Corporation, Armonk, NY, USA). Continuous variables were presented as means ± SD values, and categorical variables were presented as proportions. Differences in continuous variables were analyzed using Student *t*-tests, while chi-square tests were used to analyze categorical variables. Correlation analysis was conducted using Spearman’s correlation analysis. The factors associated with ER were also evaluated via binary logistic regression. We compared the cumulative incidence of all-cause mortality between ER and LR groups via a log-rank test. Cox’s hazard proportion analysis was used to estimate the hazard ratios (HRs) for all-cause mortality. A logistic regression analysis was used to evaluate the risk of 90-day mortality according to the timing of nephrology referral. P *<* 0.05 was considered statistically significant.

## Results

### Characteristics of patients according to timing of nephrology referral

The clinical characteristics of 820 elderly patients with incident hemodialysis are summarized in Table 1. Participants’ mean age and the mean follow-up duration were 76.3 years old and 25.1 months, respectively. Of the 820, 60.5% were men, 65.3% had diabetes, 98.3% had HTN, 36.2% had cardiovascular disease, and patients with ER group were reported in 52.4% of the subjects. Patients were assigned to one of two groups (ER and LR) according to the time of their first encounters with nephrologists. ER group had large proportions of cardiovascular disease and a central venous catheter (CVC) at initiation of dialysis, and had higher baseline Cr and albumin relative to LR group, whereas white blood cell (WBC) count, corrected Ca level, and phosphorous level were lower.

**Table 1:** Baseline characteristics of patients at initiation of dialysis according to the timing of nephrology referral.

	All 820	Early referral 430	Late referral 390	P-value
**Male (%)**	496 (60.5)	252 (58.6)	244 (62.6)	0.253
**Age, years**	76.3 ± 5.1	76.5 ± 5.3	76.2 ± 4.9	0.501
**Diabetes mellitus (%)**	523 (65.3)	288 (67.3)	235 (63.0)	0.207
**Hypertension (%)**	803 (98.3)	422 (98.4)	381 (98.2)	1.000
**Cardiovascular disease (%)**	297 (36.2)	178 (41.4)	119 (30.5)	0.001
**Malignancy (%)**	109 (13.3)	61 (14.2)	48 (12.3)	0.471
**Vascular access for the first dialysis** *				<0.001
** AVF/AVG**	197 (187/10)	150 (145/5)	47 (42/5)	
** CVC**	598	276	322	
**Systolic pressure (mmHg)**	130.8 ± 25.6	130.1 ± 25.7	131.6 ± 25.5	0.395
**Diastolic pressure (mmHg)**	73.4 ± 14.8	72.9 ± 15.0	74.1 ± 14.5	0.253
**Creatinine (mg/dL)**	6.02 ± 2.47	6.20 ± 2.35	5.83 ± 2.58	0.033
**eGFR (ml/min/1.73m** ^**2**^ **)** ^**†**^	10.48 ± 4.86	9.88 ± 4.37	11.15 ± 5.26	<0.001
**WBC (/mm** ^**3**^ **)**	9.4 ± 5.6	8.3 ± 4.2	10.7 ± 6.5	<0.001
**Hemoglobin (g/dL)**	9.5 ± 1.7	9.5 ± 1.6	9.4 ± 1.7	0.483
**Albumin (g/dL)**	3.2 ± 0.6	3.4 ± 0.6	3.0 ± 0.6	<0.001
**Corrected Ca (mg/dL)**	8.9 ± 0.8	8.8 ± 0.7	9.0 ± 0.9	0.003
**P (mg/dL)**	5.0 ± 1.7	4.9 ± 1.6	5.2 ± 1.9	0.012
**Follow up duration, months**	25.1 ± 28.1	27.5 ± 26.1	22.5 ± 30.0	0.012

Abbreviations: AVF, arteriovenous fistula; AVG, arteriovenous graft; CVC, central venous catheter; eGFR, estimated glomerular filtration rate; WBC, white blood cell

* Vascular access was analyzed in 795 patients because of unavailable data.

^†^eGFR was calculated using the Modification of Diet in Renal Disease study equation

### Factors associated with early referral

Higher Cr (OR = 1.104, P = 0.007), higher albumin (OR = 2.087, P < 0.001), lower corrected Ca (OR = 0.769 P = 0.010) and lower phosphorous (OR = 0.902, P = 0.045), and arteriovenous fistula (AVF)/arteriovenous graft (AVG) rather than CVC (OR = 3.371, P < 0.001) were observed in the ER group relative to the LR group (Table 2).

**Table 2:** Factors associated with early referral using multivariable logistic regression.

	Odds ratio	95% CI	P-value
**Male/Female**	0.809	0.588–1.114	0.194
**Age at diagnosis**	1.031	1.000–1.063	0.050
**Creatinine (mg/dL), per 1- unit** ↑	1.104	1.027–1.187	0.007
**Albumin (g/dL), per 1- unit** ↑	2.087	1.598–2.725	<0.001
**Corrected Ca (mg/dL), per 1- unit** ↑	0.769	0.630–0.938	0.010
**P (mg/dL), per 1- unit** ↑	0.902	0.816–0.998	0.045
**AVF+AVG/CVC for the first dialysis**	3.371	2.252–5.046	<0.001

Abbreviations: AVF, arteriovenous fistula; AVG, arteriovenous graft; CVC, central venous catheter

### Long-and short-term all-cause mortality according to the referral timing

**Fig 1:**
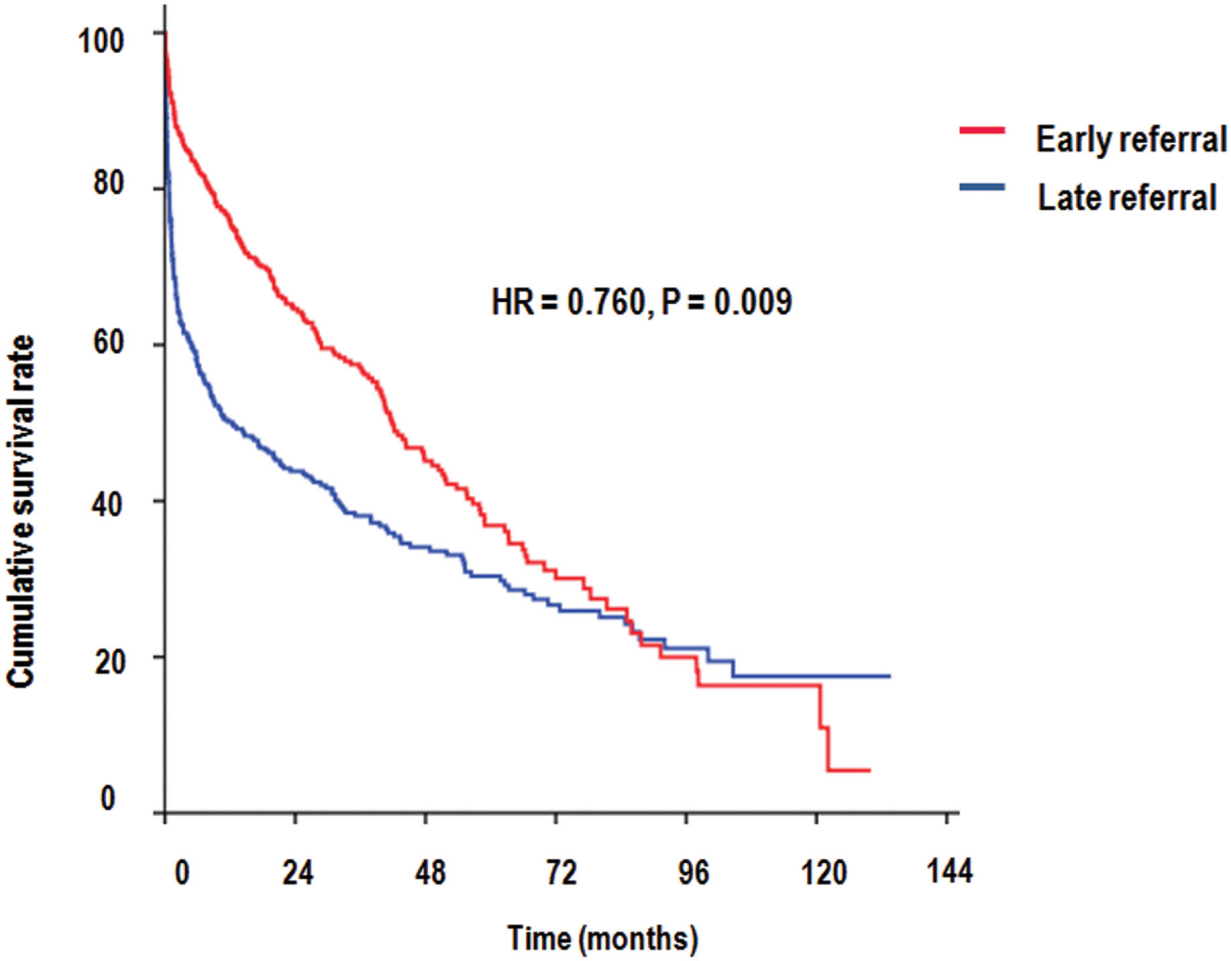
Kaplan—Meier curves for 820 patients with end stage renal disease who initiated hemodialysis during total follow-up period. All-cause mortality rates according to the referral timing.

**Fig 2:**
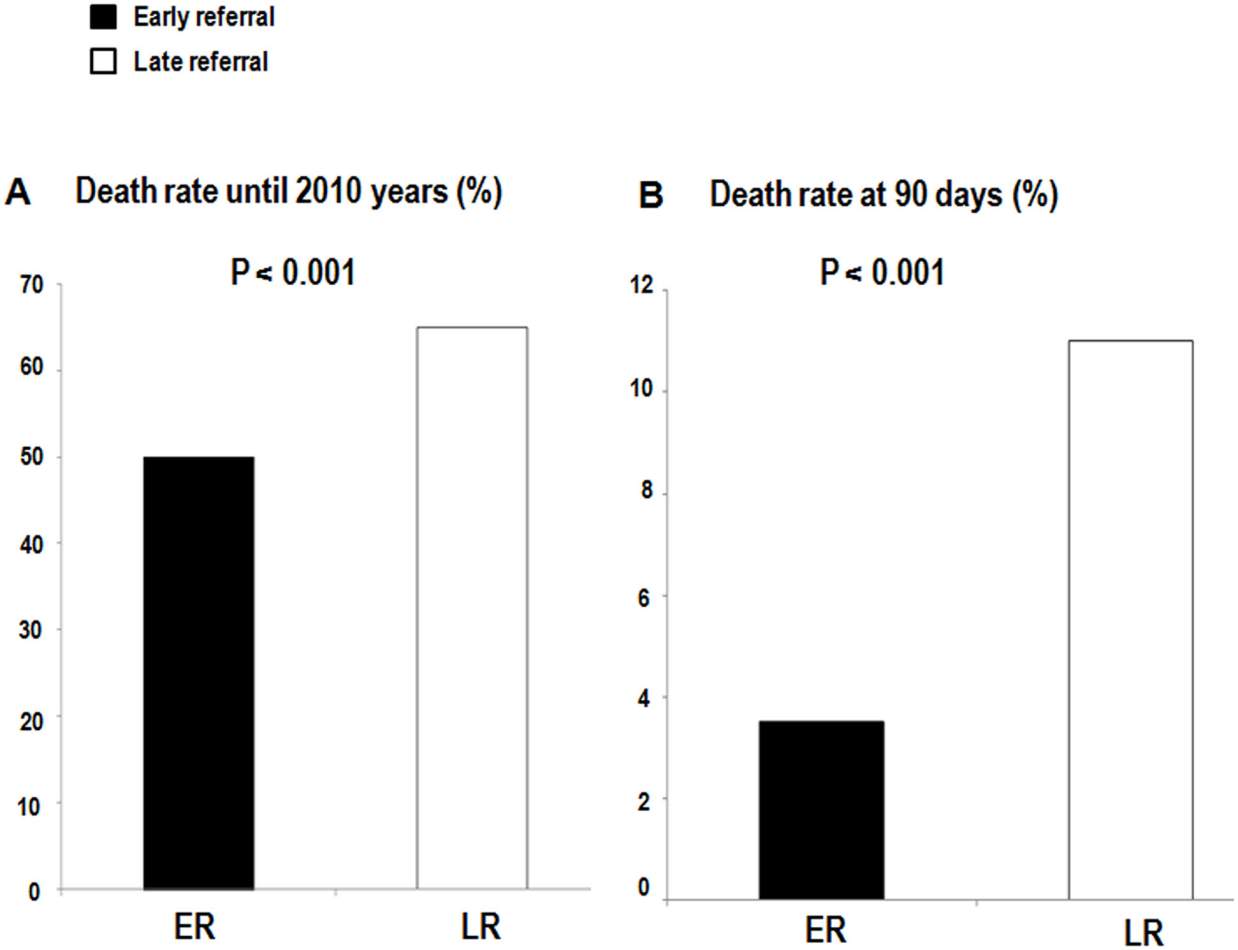
Death rate during total follow-up period (A) and at 90 days (B) according to referral timing.

During a mean follow-up period of 25.1 months, 213 patients (49.5%) in the ER group and 253 patients (64.9%) in the LR group, respectively, died (Figs 1 and 2A). In an adjusted model, the ER group displayed a 24% reduced risk of mortality relative to the LR group (HR = 0.760, P = 0.009) (Table 3). Old age, LR, CVC, high WBC count and corrected Ca level, and lower levels of albumin, Cr, hemoglobin, and sodium were significantly (P < 0.05) associated with increased risk of mortality (data not shown).

**Table 3:** Effect of timing of nephrology referral on patient survival using multivariable cox proportional hazard model.

Cox proportional hazard model	HR ER/LR	95% CI	P-value
**Total follow up ACM**	0.760	0.619–0.933	0.009
**90-Day ACM**	0.422	0.215–0.828	0.012
**After 90-Day ACM**	1.156	0.879–1.521	0.301

Abbreviations: ER, early referral; LR, late referral; ACM, all-cause mortality Multivariable, adjusted for age, gender, diabetes mellitus, hypertension, serum creatinine, albumin, comorbidites including cardiovascular disease and malignancy, type of vascular access at the first dialysis

The overall 90-day mortality rates for patients with ESRD were 3.5% (15 of 430) in the ER group and 11% (43 of 390) in the LR group (Fig 2B). The ER group also demonstrated a reduced 90-day mortality rate relative to that of the LR group after adjustment for age, gender, DM, HTN, cardiovascular disease, malignancy, type of vascular access at the time of the first dialysis, WBC, albumin, and Cr (HR = 0.422, P = 0.012) However, after 90 days, the referral pattern was not associated with the mortality rates (Table 3). High WBC, presence of cardiovascular disease at start of dialysis were significantly (P < 0.05) associated with increased risk of mortality (data not shown).

## Discussion

In this study of patients initiating dialysis between 2000 and 2010, 820 patients aged 70 years or older were included, and 52.4% presented early nephrology referral. We identified the factors associated ER and found ER also improved short-and long-term survival rates of the elderly.

The proportion of patients who received LR was higher in our study population compare to the 41% reported in a study conducted by Kim et al. showing an extended cutoff point for LR (LR < 1 year) [16]. In general, approximately 30% of patients globally [8,17], 34% of patients in the United States and United Kingdom, and approximately 23% of patients in Australasia [17] receive LR. The possible explanations for the difference observed in our study compared to others are as follows: The first reason may be that the study was performed in a large tertiary hospital in Korea, in which individuals are at higher risk of LR. The second possible reason is that there was no universal definition for nephrology referral timing in CKD patients. The definitions for LR timing were within 1 month [18], 3–4 months [19–27], 6 months [28,29], and 1 year [16]. Although some studies have questioned whether a cutoff time of 3 months within which to distinguish between LR and ER is adequate [16,30], the most broadly accepted definition of LR is the first encounter with a nephrologist within 3–4 months prior to ESRD diagnosis [8,17]. We adopted this period in this study.

The factors associated with ER were higher albumin and Cr levels, and higher frequency of AVF/AVG. With the exception of the finding that higher Cr level was observed in the ER group, our results for factors associated with ER were consistent with those of Arora et al [31].

A reduced long-term mortality rate of 24% was observed in the ER group. In our data, a steeper slope of mortality was observed during the early-period (within 90-days) of dialysis and was remarkable in the LR group. After 90 days, the slope of mortality ran parallel between the ER and LR groups (Fig 1). To highlight the effect of referral timing on mortality within 90 days, we removed 90-day mortality data and calculated the mortality rate after 90 days. The 90-day mortality reduction in ER patients was 58% for patients aged 70 years or older; this reduction was greater than those observed during the total follow-up period. Furthermore, the statistical significance of the mortality differences after 90 days between the ER and LR groups was not observed across age groups. Therefore, mortality differences for the total follow-up period were related to the early-period (within-90 days) of dialysis. These results were consisted with a recent systematic review reporting that ER reduced mortality compared with LR, and differences in mortality at 5 years were correlated with the initial 0–3 month mortality rates [8]. In addition, some reports showed that patients who had undergone incident hemodialysis had the highest mortality in the first several months after dialysis initiation and pre-dialysis care was included as a modifiable risk factor [32–36]. In fact, previous studies reported that ER patients had higher survival rate in elderly patients as well as younger patients [12,16]. However, in the elderly, whether timing of referral is associated with long- and short-term (90-day) mortality has not been reported. Therefore, we emphasize the originality of this study in that timing of referral affects the early-period (90-day) mortality and long-term mortality of the elderly. LR group, higher WBC count, presence of cardiovascular disease at start of dialysis associated with short-term mortality. LR group, old age, CVC, higher WBC count and lower levels of albumin, hemoglobin, and sodium were significantly associated with increased risk of long-term mortality. Although we did not specifically explain the contributory causes of death in our data, it was inferred that acute complications during the early period of dialysis, including infection, vascular access, and cardiovascular events, could contribute to higher early mortality rates such as those reported in the U.S. Renal Data System [37]. Therefore, ER could lead to planned transition to ESRD by preventing acute complications and reducing mortality, which is also true in elderly patients.

In fact, previous studies showing the influence of the timing of referral on mortality rates have demonstrated negative results. Schmidt et al. reported no statistical difference in 4-month mortality between ER (>1 month) and LR [18]. In addition, Roubicek et al. showed that there were no differences in the 3-month, 1-year, or 5-year survival rates according to referral patterns (ER > 4 months) in 270 patients [21]. Wolfgang et al. reported no material improvement in 1-year survival rates despite significant trends toward ER in older patients [13]. There are several limitations to the studies showing negative results as follows: 1-month period before dialysis is too short to demonstrate the benefits of nephrological intervention, and the studies involved small study populations, and did not perform direct comparisons between ER and LR to evaluate survival outcomes but examined trends in 1-year survival rates over a decade in the elderly.

This study has several limitations. First, although favorable biochemical parameters for hemoglobin and albumin were postulated as the cause of improved survival rates, we cannot explain exactly which interventions, performed by nephrologists for the ER group, improved survival rates. Second, we did not adjust for medications.

In conclusion, timely referral was also associated with reduced mortality in the elderly with incident hemodialysis treatment; in particular, the initial 90-day mortality reduction in ER patients contributed to mortality differences observed for the total follow-up period.
